# Hybridization capture increases on-target nanopore sequencing of plant RNA tobamovirus- derived cDNA libraries

**DOI:** 10.3389/fmicb.2026.1925769

**Published:** 2026-08-31

**Authors:** Andres S. Espindola, Francisco Mosquera-Yuqui, Francisco Ochoa-Corona, Stephen Marek

**Affiliations:** 1 Institute for Biosecurity and Microbial Forensics, Oklahoma State University, Stillwater, OK, United States; 2 Department of Entomology and Plant Pathology, Oklahoma State University, Stillwater, OK, United States

**Keywords:** biosecurity, ds-cDNA, high-throughput sequencing, method development, oxford nanopore, plant RNA tobamovirus, plant virus surveillance, target capture

## Abstract

High-throughput sequencing (HTS) can support plant virus surveillance, but host nucleic acids often reduce on-target read recovery. We evaluated a targeted hybridization-capture workflow in which barcoded double-stranded cDNA (ds-cDNA) libraries generated from plant RNA extracts spiked with lyophilized tobamovirus-positive controls were enriched before Oxford Nanopore sequencing. Biotinylated probes targeted conserved regions of cucumber green mottle mosaic virus (CGMMV), species *Tobamovirus viridimaculae*; pepper mild mottle virus (PMMoV), species *Tobamovirus capsici*; and tobacco mosaic virus (TMV), species *Tobamovirus tabaci*. Across four pairs per virus, relative target-read abundance increased after capture from 0.76 ± 0.33% to 37.62 ± 15.72% for CGMMV, 8.16 ± 3.86% to 24.68 ± 12.34% for PMMoV, and 15.62 ± 10.40% to 36.83 ± 30.33% for TMV. Exact two-sided Wilcoxon signed-rank tests yielded *P* = 0.125 for each virus; with four nonzero differences in a common direction, this was the minimum attainable two-sided *P* value. Genome-coverage breadth was maintained. Retrospective duplex qPCR supported an increased virus-to-18S ratio for CGMMV, showed a variable PMMoV response, and showed a decreased virus-to-18S ratio for TMV because the 18S signal shifted earlier by as much as or more than the TMV signal. The findings provide proof-of-concept evidence for target-dependent library enrichment but do not establish analytical sensitivity, diagnostic performance, or field validity. Validation with naturally infected, low-titer, and mixed-infection samples and comparison with simpler targeted workflows are required.

## 1 Introduction

High-throughput sequencing (HTS) is increasingly used in plant virology because it can recover information from multiple agents in a single sequencing run and can support surveillance, quarantine, and certification workflows (Maina et al., 2024). However, shotgun sequencing of plant nucleic acids can be dominated by host-derived molecules, reducing the proportion of informative pathogen reads and increasing sequencing cost (Farrall et al., 2024; Pilling et al., 2024). For targeted surveillance of known viruses, one possible strategy is to increase the representation of target-derived nucleic acids prior to sequencing while retaining sufficient sequence information to assess identity and genome coverage.

Oxford Nanopore sequencing offers practical advantages for decentralized or time-sensitive workflows, including portable instrumentation, flexible run length, and generation of long reads (Farrall et al., 2024; Maina et al., 2024). Because short-read platforms and PCR-based assays remain efficient for many applications, targeted long-read workflows are best evaluated for defined use cases, such as rapid local sequencing, multiplexed sample processing, recovery of longer target-derived molecules, and enrichment of known targets from complex backgrounds.

Tobamoviruses are rigid rod-shaped, positive-sense single-stranded RNA viruses with genomes of approximately 6.3–6.8 kb (Lewandowski, 2008). They are transmitted readily through mechanical contact and, in some cases, by seed-associated contamination, which contributes to their persistence in production systems and their relevance to biosecurity programs (Giesbers et al., 2023; Lewandowski, 2008; Salem et al., 2016). Cucumber green mottle mosaic virus (CGMMV), species *Tobamovirus viridimaculae*; pepper mild mottle virus (PMMoV), species *Tobamovirus capsici*; and tobacco mosaic virus (TMV), species *Tobamovirus tabaci*, are representative tobamoviruses affecting cucurbit, pepper, and solanaceous crops, respectively (Ainsworth, 1935; Ishibashi et al., 2023; Kenyon et al., 2014; Tian et al., 2023). Current ICTV binomial species names are provided at first mention together with the corresponding traditional virus names and abbreviations; the abbreviations CGMMV, PMMoV, and TMV are used thereafter for readability. Hybridization capture with biotinylated probes and streptavidin-coated magnetic beads has improved recovery of low-abundance viral sequences in human and vertebrate systems (Briese et al., 2015; Dickson et al., 2021). In plant virology, however, probe-based capture remains less explored, particularly for plant RNA virus libraries prepared for long-read sequencing (Farrall et al., 2024). Because probe capture is inherently target biased, it is not appropriate as a stand-alone strategy for novel-virus discovery. Its potential value is instead in targeted panels where the viruses of interest are known in advance and the goal is to increase on-target sequence recovery from plant-dominated nucleic acid backgrounds.

Here, we evaluated hybridization-based capture as a targeted pre-sequencing step to increase recovery of tobamovirus-derived ds-cDNA molecules from barcoded libraries. Healthy plant RNA extracts spiked with lyophilized positive tissue controls provided a controlled plant RNA background for matched pre-capture and post-capture comparisons. We designed 5′-biotinylated probes from conserved regions of CGMMV, PMMoV, and TMV and assessed changes in relative viral read abundance, breadth of genome coverage, and the ratio of viral qPCR signal to a host-derived 18S marker. This controlled experimental system was used to assess workflow feasibility and identify parameters for future validation with naturally infected and low-titer samples.

## 2 Materials and methods

### 2.1 Study design

**FIGURE 1:**
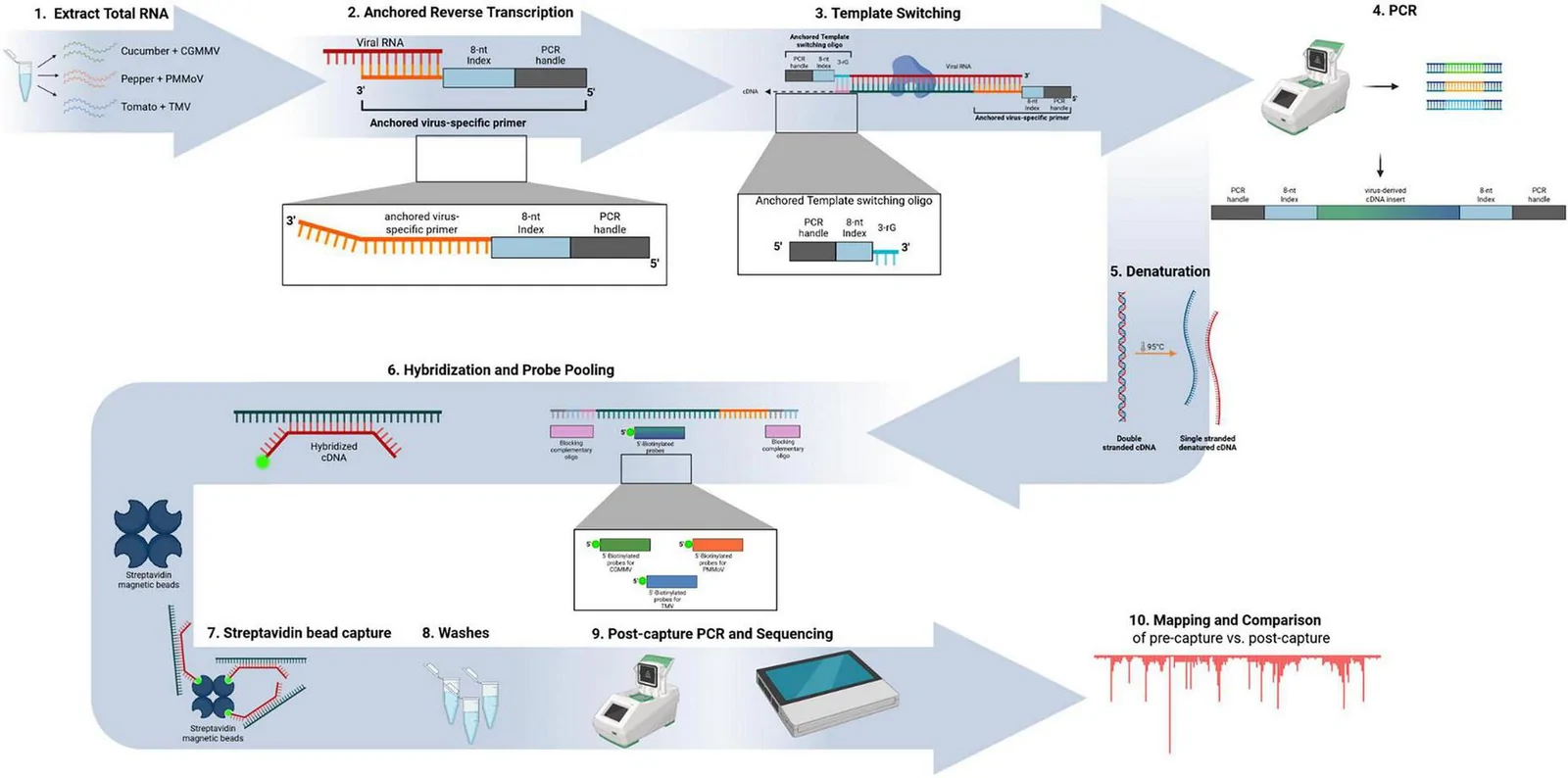
Template-switching cDNA synthesis and hybridization-based capture workflow for tobamovirus-derived ds-cDNA libraries. Total RNA was extracted from cucumber, bell pepper, and tomato tissue backgrounds spiked with lyophilized positive controls for CGMMV, PMMoV, and TMV. Anchored virus-specific primers introduced an 8-nt index and PCR handle during reverse transcription. Template-switching oligonucleotides introduced the opposite PCR handle and index. Amplified ds-cDNA libraries were denatured, hybridized with 5′-biotinylated probes and blocking oligonucleotides, captured on streptavidin-coated magnetic beads, washed, re-amplified, sequenced, and compared before and after capture. Created in BioRender. Espindola, A. (2026) https://BioRender.com/l4agd1q.

This study used a paired design to compare pre-capture and post-capture barcoded ds-cDNA libraries for three tobamoviruses (Figure 1). Healthy plant RNA extracts were spiked with lyophilized positive tissue controls and processed through anchored reverse transcription, template switching, PCR amplification, hybridization capture, and Oxford Nanopore sequencing. The paired libraries allowed the capture step to be evaluated while holding the starting material and upstream library preparation workflow constant. Four replicate pairs per virus-host combination were used for the final sequencing comparison. The study was designed as an initial workflow-feasibility experiment rather than as a powered validation study. Additional retained ds-cDNA aliquots were analyzed retrospectively by duplex qPCR to independently evaluate changes in target-to-host signal following capture.

### 2.2 Oligonucleotide design

To enable multiplex processing, both reverse primers and template-switching oligonucleotides carried a shared 5′ anchor comprising an 8-nt sample barcode and a common PCR amplification sequence. The anchored reverse primers also contained a virus-complementary region at the 3′ end, whereas template-switching oligonucleotides contained terminal riboguanosines to support template switching. Anchored reverse primers, template-switching oligonucleotides, blocking oligonucleotides, and 5′-biotinylated capture probes are provided in Supplementary Tables 1, 2.

Capture probes were designed from multiple sequence alignments of complete genome sequences available in the NCBI Virus database through April 2024. For CGMMV, PMMoV, and TMV, 161, 48, and 94 complete genomes were aligned, respectively. Conserved regions were selected for synthesis of 5′-biotinylated probes. Probe names, target coordinates, GC content, and melting temperatures are summarized in Supplementary Table 1.

### 2.3 Positive controls, plant materials, and RNA extraction

Lyophilized positive tissue controls derived from naturally infected tissue were obtained for CGMMV, PMMoV, and TMV (Agdia, Elkhart, IN, USA). Virus-specific hydrolysis-probe assays and an initial NAD5 plant control assay were designed or adapted to verify RNA recovery from the spiked extracts (Supplementary Table 3) (Botermans et al., 2013; Menzel et al., 2002). During later validation of retained ds-cDNA libraries, four candidate host-control assays (ACTIN, GAPDH, EF1α, and 18S) were screened for detectable and reproducible amplification and compatibility with the virus-specific duplex assays. The 18S assay was selected pragmatically because it provided the most consistent detectable host-derived signal across the retained libraries (Aslam et al., 2009). Because capture was expected to alter the viral-to-host composition of the libraries, 18S was used as a host-derived marker rather than as a reference assumed to remain constant between pre-capture and post-capture samples. The final duplex qPCR assays therefore used 18S rather than NAD5 as the host marker (Supplementary Table 3).

Leaves from cucumber (*Cucumis sativus* L.), bell pepper (*Capsicum annuum*), and tomato (*Solanum lycopersicum*) plants 7–9 weeks old were collected from the Greenhouse Learning Center at Oklahoma State University (Stillwater, OK, USA) and transported to the laboratory on ice. Total RNA was extracted with the NucleoSpin RNA Plant kit (Macherey-Nagel, Düren, Germany). Briefly, 100 mg of tissue was ground under liquid nitrogen, suspended in 350 μL lysis buffer plus 3.5 μL beta-mercaptoethanol, and the lysate was added to one vial of the appropriate lyophilized positive control. After a 3 min incubation to allow rehydration, the mixture was homogenized by pipetting and the remaining extraction steps were completed according to the manufacturer’s instructions.

### 2.4 Barcoded ds-cDNA synthesis

For each replicate library, 4 μL of total RNA (approximately 120 ng), 1 μL of the corresponding anchored reverse primer (10 μM), and 1 μL of dNTPs (10 μM) were incubated at 65°C for 5 min and chilled on ice for approximately 3 min. Reverse transcription and template switching were performed by combining the annealed RNA with 2.5 μL of template-switching RT buffer (1×), 1 μL of template-switching RT enzyme mix, and 0.5 μL of the corresponding barcode-template-switching oligonucleotide (75 μM). Reactions were incubated at 42°C for 90 min followed by 85°C for 5 min.

Switched cDNA was amplified with 1 μL of amplification primer (10 μM), 25 μL of NEBNext High-Fidelity 2× PCR Master Mix (New England Biolabs, Ipswich, MA, USA), 14 μL of nuclease-free water, and the reverse-transcription product. PCR cycling conditions were 98°C for 45 s; 30 cycles of 98°C for 10 s, 62°C for 15 s, and 72°C for 3.5 min; followed by 72°C for 5 min. Amplified ds-cDNA was cleaned with AMPure XP beads at 0.8× sample volume, quantified with the QuantiFluor ONE dsDNA system (Promega, Madison, WI, USA), and submitted for fragment analysis on an Agilent TapeStation instrument.

### 2.5 Hybridization capture of ds-cDNA libraries

Barcoded ds-cDNA libraries were captured with the xGen Hybridization Capture Core Reagents v2 kit (Integrated DNA Technologies, Coralville, IA, USA) using a modified tube protocol for long-fragment capture. For each reaction, 230 ng of library DNA was combined with 5 μL UltraPure salmon sperm DNA solution (1 mg/mL; Invitrogen, Waltham, MA, USA), 4 μL of anchored reverse primer (100 μM), and 4 μL of the corresponding forward blocking oligonucleotide (100 μM). Samples were dried in a SpeedVac concentrator and stored at −20°C for up to 2 days before capture.

Dried libraries were reconstituted in hybridization master mix, incubated at room temperature for 5–10 min, denatured at 95°C for 7 min. Four microliters of the corresponding virus-specific pool of 5′-biotinylated probes was then added to each reaction. The probe pools were prepared to target approximately 200 amol of each probe per μL of hybridization reaction. The same probe-pool volume and preparation strategy were used across reactions, but probe amount and per-probe stoichiometry were not independently optimized for CGMMV, PMMoV, or TMV. Hybridization was performed at 65°C for 18–20 h. Hybridized libraries were transferred to preconditioned Dynabeads M-270 Streptavidin beads (Thermo Fisher Scientific, Waltham, MA, USA) and incubated at 65°C for 45 min with intermittent shaking. Heated and room-temperature washes were followed by on-bead amplification using Takara LA Taq DNA Polymerase Hot-Start Version (Takara Bio, Shiga, Japan).

For the on-bead amplification step, two PCR reactions were assembled for each captured library. Each reaction contained 25 μL of resuspended beads, 10 μL LA PCR buffer (10×), 12 μL dNTPs (2.5 mM each), 4.4 μL amplification primer (100 μM), 0.6 μL LA Taq HS polymerase, and nuclease-free water to 100 μL. Cycling conditions were 95°C for 2 min; 30 cycles of 98°C for 20 s, 62°C for 15 s, and 68°C for 7 min; followed by 68°C for 6 min. Paired PCR reactions were pooled and purified with AMPure XP beads at 0.6× sample volume.

### 2.6 Duplex qPCR validation of pre-capture and post-capture libraries

Aliquots of retained pre-capture and post-capture ds-cDNA libraries were later analyzed by duplex qPCR to provide an orthogonal check of whether capture changed viral signal relative to a host-derived ds-cDNA signal. Reactions were prepared with Luna Universal Probe qPCR Master Mix (New England Biolabs, Ipswich, MA, USA) in 20-μL volumes containing 10 μL 2× master mix, 0.8 μL each of the virus-specific forward and reverse primers (10 μM), 0.8 μL each 18S primer (10 μM), 0.4 μL each of the virus-specific and 18S hydrolysis probes (10 μM), 1.0 μL ds-cDNA, and nuclease-free water to volume. Reactions were run on a Rotor-Gene instrument according to the manufacturer’s recommended Luna hydrolysis-probe cycling conditions. Virus-specific oligonucleotides were CGMMVfm7/CGMMVby77/CGMMV-P1, PMMoVfm10/PMMo Vby10/PMMoV-P1, and TMVfm5/TMVby55/TMV-P1; 18SF/ 18SR/18S-Pfm1 was used as the host-derived marker assay (Supplementary Table 3). Each duplex assay was calibrated with a six-point 10-fold dilution series (0.05 to 5 × 10–7 ng/μL) of a synthetic gBlock containing the corresponding viral amplicon and the 18S target region.

CGMMV was evaluated using five paired libraries, PMMoV using six paired libraries, and TMV using four paired libraries. Each duplex assay was evaluated using a six-point, 10-fold dilution series to assess linearity and amplification efficiency for the virus-specific and 18S channels. Ct values were converted to relative target quantities using the corresponding virus-specific and 18S standard curves. For each library, a virus-to-18S relative quantity ratio was calculated, and the paired post-capture/pre-capture fold change in this ratio was used to describe how the viral target changed relative to the assayed 18S locus. A fold change greater than 1 indicates an increased viral-target-to-18S ratio after capture, whereas a value below 1 indicates a decreased viral-target-to- 18S ratio.

Raw virus and 18S Ct shifts, △*Ct*(Ct_virus_−Ct_18S_), and △△*Ct*(△Ct_post_−△Ct_pre_) were also examined descriptively to evaluate the direction of change in both assay channels. Because assay efficiencies differed from 100%, the reported virus-to-18S fold changes were based on standard-curve-derived concentration ratios rather than an uncorrected 2^−△△Ct^ calculation. The qPCR analysis was retrospective and used a single 18S amplicon as a proxy for host-derived ds-cDNA. Accordingly, the qPCR results were interpreted as complementary measurements of virus-to-18S behavior, not as gene-expression normalization or as an independent estimate of total host-background depletion.

### 2.7 Oxford nanopore sequencing

Pre-capture and post-capture amplicons were sequenced with the Ligation Sequencing Kit V14 (SQK-LSK114; Oxford Nanopore Technologies, Oxford, UK) on an R10.4.1 MinION flow cell (FLO-MIN114). The volume of each library required to prepare a 200 fmol sequencing pool was calculated from DNA concentration and the average library size estimated by fragment analysis; summary size estimates are provided in Supplementary Table 4.

Pooled libraries underwent end repair and dA-tailing with the NEBNext Ultra II End Prep system (New England Biolabs, Ipswich, MA, USA), followed by a 1× AMPure XP cleanup, adapter ligation, and a final 0.4× bead cleanup using Short Fragment Buffer to retain a broad fragment-size distribution. A total of 75 fmol of the final pooled library was loaded onto the flow cell.

### 2.8 Bioinformatic processing and statistical analysis

Raw FASTQ files generated by MinKNOW were basecalled with Dorado v0.6.1 using the sup model. Reads with Q-scores ≥ 9 were demultiplexed with the match subcommand of the demultiplexer package by matching the 31-nt barcode-containing index sequence with one allowed mismatch. Duplicate read identifiers were removed with an AWK script. Reads were then filtered with Filtlong v0.2.1 (minimum length 200 nt, maximum length 8000 nt, top 99% by quality) and trimmed with Cutadapt to remove the first 120 nt from the 5′ end (Martin, 2011).

Filtered reads were mapped separately to the corresponding RefSeq genome for each target with Minimap2 using the map-ont preset (Li, 2021). SAM files were processed with SAMtools to remove unmapped reads, convert to BAM format, sort, and index (Danecek et al., 2021). Relative viral abundance was calculated as the proportion of reads mapping to the target genome divided by total reads for each library. Genome coverage was calculated from the mapping output as the percentage of genome positions covered by at least one mapped read. The matched library pair was the unit of analysis, and each virus was analyzed separately. Post-capture and pre-capture relative abundance and genome coverage were compared using exact two-sided Wilcoxon signed-rank tests implemented by complete sign enumeration in Python 3.10.12. Zero differences were discarded, and tied absolute differences were assigned midranks. When every paired difference was zero, no inferential *P* value was calculated. Paired *t*-tests were retained only as secondary analyses. Descriptive reporting included the raw paired values, mean ± SD, paired fold-change range when defined, and median post-minus-pre difference. With four pairs, normality of paired differences could not be assessed reliably, and exact nonparametric *P*-value resolution and power were limited. All *P* values were two-sided, and no transformation was applied.

## 3 Results

### 3.1 Fragment analysis of barcoded libraries

Fragment analysis showed depletion of short fragments after hybridization capture for all three virus-host combinations. Estimated average library sizes ranged from 1.1 to 2.3 kb in pre-capture libraries and from 1.2 to 2.8 kb in post-capture libraries (Supplementary Table 4).

### 3.2 Observed paired increases in on-target read proportions

**FIGURE 2:**
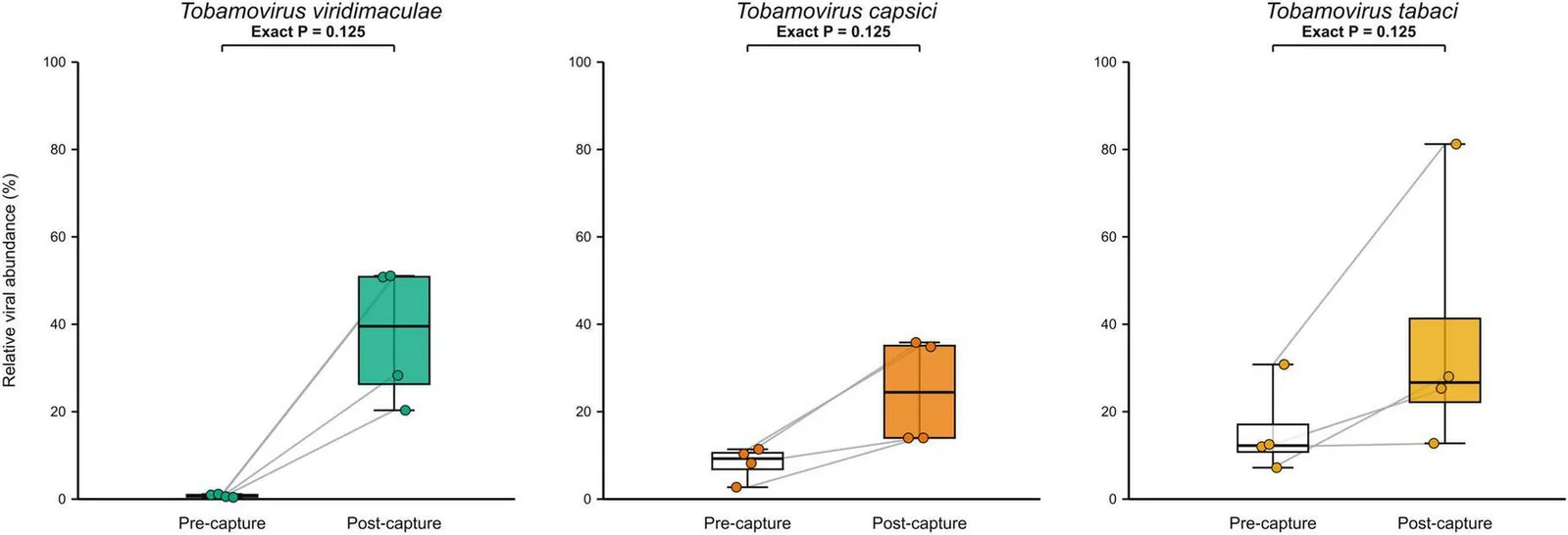
Relative viral abundance in paired pre-capture and post-capture libraries for CGMMV (left), PMMoV (center), and TMV (right). Each point and gray connecting line represents one retained matched library pair (*n* = 4 per virus); boxplots summarize the distribution. Panel annotations show exact two-sided Wilcoxon signed-rank *P* values. The matched library pair, not individual reads, was the unit of analysis.

Relative viral abundance was compared between paired pre-capture and post-capture libraries (Figure 2; Table 1). For CGMMV, all four pairs increased after capture, with mean relative abundance rising from 0.76 ± 0.33% to 37.62 ± 15.72%. Pairwise enrichment ranged from 45.16-fold to 53.78-fold, and the median paired difference was 38.79 percentage points. The exact Wilcoxon signed-rank test yielded *P* = 0.125; the secondary paired *t*-test analysis yielded *P* = 0.017.

**TABLE 1:** Summary of paired sequencing outcomes after hybridization capture.

Virus	Relative abundance, pre → post (mean ± SD, %)	Median paired difference (percentage points)	Paired fold-change range	Exact Wilcoxon P (relative abundance)	Genome coverage, pre → post (mean ± SD, %; median Δ)	Exact Wilcoxon P (coverage)
CGMMV	0.76 ± 0.33 → 37.62 ± 15.72	38.79	45.16–53.78	0.125	78.81 ± 17.17 → 71.16 ± 6.46; −4.66	0.625
PMMoV	8.16 ± 3.86 → 24.68 ± 12.34	17.37	1.71–5.14	0.125	100.00 ± 0.00 → 100.00 ± 0.00; 0.00	Not testable
TMV	15.62 ± 10.40 → 36.83 ± 30.33	16.82	1.06–3.90	0.125	99.88 ± 0.19 → 98.93 ± 0.66; −1.07	0.125

*n* = 4 matched library pairs per virus. Differences were calculated as post-capture minus pre-capture. Reported *P* values are from two-sided exact Wilcoxon signed-rank tests. Secondary paired *t*-test results are provided for comparison: CGMMV, *P* = 0.017 for relative abundance and *P* = 0.352 for genome coverage; PMMoV, *P* = 0.040 for relative abundance, with genome coverage not testable because all paired differences were zero; and TMV, *P* = 0.139 for relative abundance and *P* = 0.054 for genome coverage.

For PMMoV, all four pairs increased after capture, from 8.16 ± 3.86% to 24.68 ± 12.34% on average. Pairwise enrichment ranged from 1.71-fold to 5.14-fold, the median paired difference was 17.37 percentage points, the exact Wilcoxon *P* value was 0.125, and the secondary paired *t*-test analysis *P* value was 0.040. For TMV, all four pairs also increased, from 15.62 ± 10.40% to 36.83 ± 30.33% on average, but the magnitude varied substantially among pairs (1.06-fold to 3.90-fold). The median paired difference was 16.82 percentage points; the exact Wilcoxon *P* value was 0.125 and the secondary paired *t*-test analysis *P* value was 0.139. With four nonzero paired differences in a common direction, *P* = 0.125*P* = 0.125*P* = 0.125 is the smallest attainable two-sided exact Wilcoxon signed-rank *P* value. Accordingly, the results are described as observed paired increases rather than as statistically significant increases under the primary analysis. For TMV, the increase was smaller and more variable than that observed for CGMMV and PMMoV.

### 3.3 Duplex qPCR corroborated CGMMV enrichment but showed variable PMMoV and TMV responses

All three duplex assays produced approximately linear six-point standard curves over five orders of magnitude. CGMMV and PMMoV standard curves showed R^2^ values of 0.9995–0.9998 and amplification efficiencies of 94.8–95.4% for both virus and 18S channels; the TMV assay remained linear but showed lower efficiencies (89.8–90.5%) (Supplementary Table 5).

CGMMV qPCR results agreed closely with the sequencing analysis. In all five paired libraries, post-capture samples showed earlier viral Ct values than their pre-capture counterparts, and the virus/18S concentration ratio increased 12.49–32.20-fold (median 18.13-fold) after capture (Supplementary Table 6). PMMoV showed the same direction in five of six pairs, with virus-to-18S increases of 1.25–18.32-fold (median 2.20-fold), although one pair decreased. For TMV, absolute viral Ct values were earlier in all four post-capture libraries, but the 18S Ct values also shifted earlier and by an equal or greater absolute amount in every pair. Consequently, the TMV/18S concentration ratio decreased in all four pairs (0.33–0.62-fold). Thus, the qPCR data supported an increased virus-to-18S ratio for CGMMV, provided variable support for PMMoV, and showed no increase in the TMV-to-18S ratio.

The 18S assay was not expected to remain constant across capture conditions, because successful enrichment could alter the relative abundance of viral and host-derived ds-cDNA. However, the observed direction of the 18S shift was notable: mean 18S Ct changed from 18.68 ± 0.72 before capture to 15.94 ± 1.13 after capture for CGMMV, from 20.01 ± 1.63 to 18.67 ± 2.47 for PMMoV, and from 19.51 ± 1.13 to 17.17 ± 1.44 for TMV. Thus, the assayed 18S target appeared more abundant, not depleted, after capture. Across all evaluated libraries, 18S Ct ranged from 17.68 to 19.69 before capture and 14.24 to 17.23 after capture for CGMMV; from 17.36 to 21.80 before and 14.37 to 21.20 after capture for PMMoV; and from 18.07 to 20.66 before and 15.83 to 18.97 after capture for TMV. This pattern may reflect nonspecific retention and post-capture amplification of 18S-containing molecules or locus-specific behavior of the short 18S amplicon. Therefore, the virus-to-18S ratios should be interpreted only as relative behavior of these two qPCR targets, not as a quantitative estimate of total host-background removal.

### 3.4 Breadth of genome coverage was maintained after capture

**FIGURE 3:**
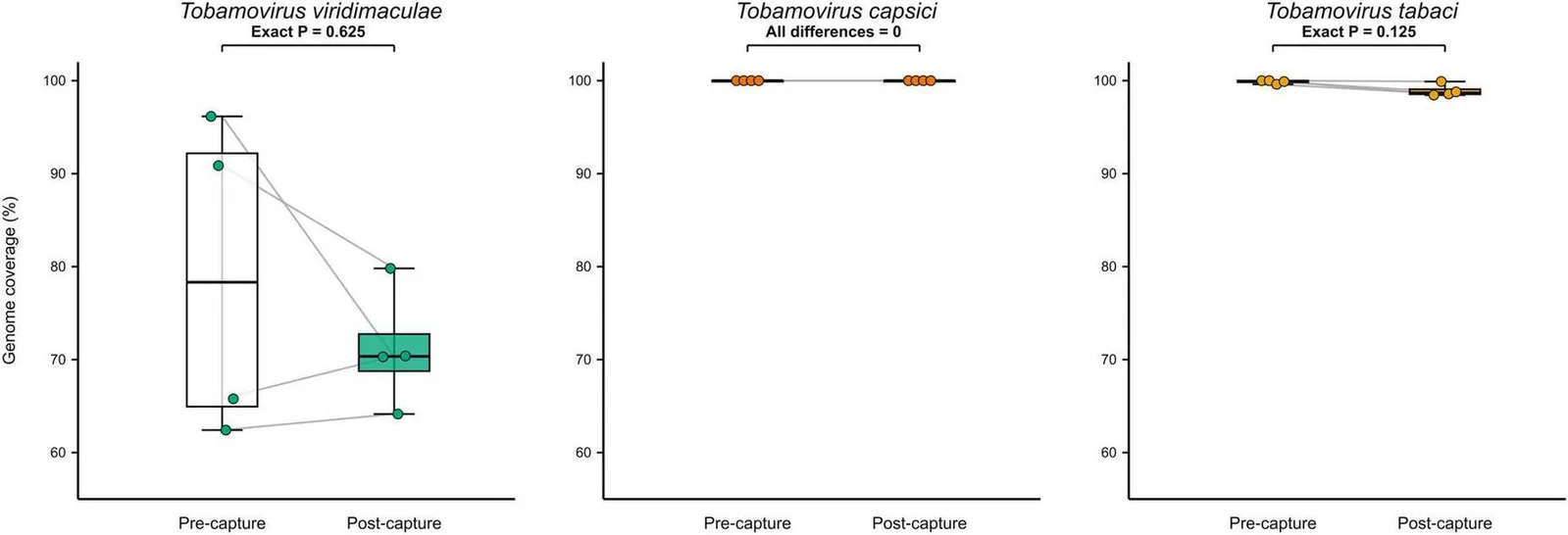
Genome coverage in paired pre-capture and post-capture libraries for CGMMV (left), PMMoV (center), and TMV (right). Each point and gray connecting line represents one retained matched library pair (*n* = 4 per virus); boxplots summarize the distribution. Panel annotations show exact two-sided Wilcoxon signed-rank *P* values. PMMoV coverage was 100% in every pre-capture and post-capture library, so all paired differences were zero and an inferential test was not informative.

Genome coverage was summarized as the percentage of each reference genome covered by mapped reads (Figure 3; Table 1). For CGMMV, mean coverage changed from 78.81 ± 17.17% before capture to 71.16 ± 6.46% after capture; the median paired difference was −4.66 percentage points, the exact Wilcoxon *P* value was 0.625, and the secondary paired *t*-test analysis *P* value was 0.352. PMMoV coverage was 100.00% in every pre-capture and post-capture library, so all paired differences were zero and an inferential test was not informative. For TMV, mean coverage changed from 99.88 ± 0.19% before capture to 98.93 ± 0.66% after capture; the median paired difference was −1.07 percentage points, the exact Wilcoxon *P* value was 0.125, and the secondary paired *t*-test analysis *P* value was 0.054. These values indicate that capture did not produce a substantial observed loss of genome-coverage breadth in the paired libraries.

## 4 Discussion

This study provides proof-of-concept evidence that hybridization-based capture can increase the representation of target-derived reads in barcoded ds-cDNA libraries prepared from plant RNA backgrounds spiked with tobamovirus-positive controls. All four pairs increased in relative abundance for each virus, but the exact primary test yielded *P* = 0.125 in every case because four nonzero changes in a common direction cannot produce a smaller two-sided exact Wilcoxon *P* value. The findings therefore support directional and effect-size patterns rather than statistically confirmed differences. The largest and most consistent change was observed for CGMMV, the target with the lowest pre-capture representation. PMMoV showed smaller increases and a more variable qPCR response. TMV increased in sequencing relative abundance but varied markedly in magnitude and lacked host-normalized qPCR support. Capture performance may depend on baseline target abundance, probe performance, and library composition, but the present design cannot distinguish the contribution of each factor.

The TMV qPCR results require separate consideration. Absolute TMV Ct values occurred earlier in all four post-capture libraries, indicating greater abundance of the TMV qPCR target after capture. However, the 18S Ct values also occurred earlier and shifted by an equal or larger absolute magnitude in every pair, causing the TMV/18S ratio to decrease. A later 18S Ct would have been consistent with depletion of the assayed host-derived target, whereas the observed earlier 18S Ct indicates that this particular 18S locus was retained and/or amplified efficiently during the post-capture workflow. Sequencing relative abundance and duplex qPCR do not measure identical quantities: sequencing reports the fraction of retained reads assigned across the TMV reference genome, whereas qPCR compares two short amplicons within retained ds-cDNA aliquots. The disproportionate 18S shift may reflect nonspecific retention and efficient post-capture amplification of abundant 18S-containing molecules, differences in amplification behavior associated with fragment length or composition, or other capture-induced changes in library composition. The relatively high pre-capture TMV abundance may also have reduced the observable dynamic range. Because no host-background sequencing or locus-specific capture experiment was performed, the present data cannot distinguish among these possibilities. The TMV result should therefore be interpreted as target- and method-dependent behavior rather than as evidence that total host background increased or that TMV was not recovered.

The workflow was designed to concentrate target-derived ds-cDNA rather than intact virus particles or native viral RNA. This distinction is central to interpretation. The observed effect reflects recovery of target-complementary library molecules after reverse transcription, PCR amplification, hybridization, and bead capture. This library-level interpretation provides a practical basis for future tests in naturally infected tissues and other plant virus surveillance contexts.

Because capture was performed after virus-specific reverse transcription and PCR, the workflow is best positioned as a targeted, sequence-generating enrichment step for known viruses. In that role, it can complement RT-PCR, amplicon sequencing, direct RNA capture, or unbiased metagenomic sequencing by increasing on-target recovery from barcoded libraries while retaining read-level information across larger portions of the viral genome. This use case may be relevant to targeted surveillance panels, quarantine-oriented follow-up sequencing, or method development for multiplex enrichment.

The use of Oxford Nanopore sequencing should also be interpreted in this specific context. Short-read sequencing can provide economical high-depth data for many plant virus applications, and compact plant virus genomes do not always require long reads for reconstruction. Nanopore sequencing remains attractive for portable or decentralized workflows, flexible run times, and rapid generation of long target-derived reads, and these advantages are application-dependent. The present study therefore supports a defined targeted long-read use case focused on enriched recovery of known viral targets from barcoded libraries.

Because tobamoviruses lack a 3′ poly(A) tail, gene-specific anchored reverse primers were used to initiate reverse transcription and simultaneously introduce barcodes and a common amplification site. Bias toward 3′-proximal regions is plausible in this system because tobamovirus infections produce abundant 3′-proximal subgenomic RNAs and full-length cDNA recovery from long RNA templates can be reduced by RNA degradation or premature termination during reverse transcription (Espindola et al., 2021; Lewandowski, 2008; Mosquera-Yuqui et al., 2025; Pan et al., 2013). These factors should be considered when adapting the workflow to other viruses or when using genome coverage as an interpretation criterion.

Fragment analysis indicated that short products were depleted after capture, but the library distributions remained broad. This broad size range may reflect a mixture of full-length and truncated cDNA molecules, which is common when long RNA targets are reverse transcribed from total RNA extracts (Pan et al., 2013). Although the current protocol used salmon sperm DNA and blocking oligonucleotides during hybridization, further optimization of blocking strategies could reduce off-target binding. Plant-derived C0t-1 DNA deserves evaluation as a host-specific blocker for future versions of the assay (Tao et al., 2003; Zwick et al., 1997). Probe-pool volume and stoichiometry were held constant across targets and were not optimized independently. Future experiments should evaluate probe amount, per-probe stoichiometry, probe number and genomic placement, hybridization duration, and wash stringency, particularly for TMV. Probe-panel development could also be streamlined using Electronic Diagnostic Nucleic Acid Analysis (EDNA) (Stobbe et al., 2013). Although the capture probes evaluated in this study were selected using multiple-sequence alignments, EDNA identifies short sequence signatures that are conserved within a defined target group while being discriminatory against related organisms and other nontarget sequences (Espindola and Cardwell, 2021). This approach can reduce reliance on manual alignment-based probe selection and facilitate the development of candidate oligonucleotides with greater predicted specificity (Bocsanczy et al., 2023; Nascimento et al., 2025; Pasha et al., 2025). EDNA-derived e-probe sequences can also serve as templates for developing physical oligonucleotides for other diagnostic formats (Geiser et al., 2023). For example, confirmatory primers developed from EDNA-derived e-probe sequences were used for detection of *Capsicum chlorosis virus* in the first report of this virus in the continental United States (Zhang et al., 2026). Similar conversion of curated e-probe signatures into biotinylated capture probes could simplify future panel development, although probe concentration, thermodynamic properties, genomic distribution, and capture performance would still require empirical optimization.

Several considerations define the scope of the present manuscript. First, RNA extracts were spiked with lyophilized positive tissue controls rather than obtained from naturally infected plants, so future testing should include the biological variation, inhibitor content, tissue distribution, and background complexity found in field samples. Second, the sample set was limited to three tobamoviruses and one host background per virus. Third, the study was designed to establish workflow feasibility at a single input condition and did not include a replicated dilution series. Available material and sequencing capacity were prioritized for evaluating matched pre-capture and post-capture behavior before undertaking analytical sensitivity testing. Consequently, the current data cannot establish analytical sensitivity, limit of detection, or the titer range over which capture provides a practical advantage. Fourth, mixed infections were not evaluated. Fifth, the qPCR evaluation was retrospective, used retained ds-cDNA rather than original RNA extracts, and relied on one 18S amplicon as a host-derived comparator. The 18S assay was not intended to remain constant after capture and cannot be assumed to represent total host-background behavior. Sixth, the four paired sequencing libraries per virus limited precision, exact P-value resolution, and assessment of the distribution of paired differences. Finally, direct comparisons with direct probe capture, multiplex RT-PCR, amplicon sequencing, or short-read HTS would help define the specific niche of the workflow.

The workflow also has a substantial operational burden. Hybridization alone required 18–20 h, followed by approximately 45 min of bead capture, multiple temperature-controlled washes, approximately 4 h of long-template post-capture PCR, cleanup, and subsequent Nanopore library preparation. The capture step therefore adds approximately 1 day of elapsed time and requires work across at least two laboratory days. For a small number of known viruses, multiplex RT-PCR followed by amplicon sequencing would generally be faster, simpler, and likely less expensive. The potential advantage of hybridization capture would need to arise from applications involving larger target panels, recovery of sequence beyond short diagnostic amplicons, or multiplex processing for which increased on-target sequencing offsets the additional capture burden. The present study did not perform a formal reagent-cost, labor, or turnaround-time comparison, and no claim of cost-effectiveness is made. Future work should include independently prepared dilution-series samples spanning low titers, negative and mixed-infection controls, optimization of probe amount and stoichiometry, and direct comparison with alternative targeted workflows.

## 5 Conclusion

Hybridization-based capture of barcoded ds-cDNA libraries produced observed paired increases in target-read relative abundance for CGMMV, PMMoV, and TMV in a controlled plant RNA background, with the largest effect for CGMMV and the most variable magnitude for TMV. Exact two-sided Wilcoxon tests yielded *P* = 0.125 for each virus, reflecting the limited resolution of four retained pairs rather than statistically confirmed differences under the primary analysis. Genome-coverage breadth was maintained, while retrospective qPCR showed an increased virus-to-18S ratio for CGMMV, a variable PMMoV response, and a decreased TMV-to-18S ratio despite earlier absolute TMV Ct values after capture. These findings establish workflow feasibility but not analytical sensitivity, diagnostic performance, cost-effectiveness, or field validity. The method requires target-specific probe optimization, dilution-series testing, naturally infected samples, mixed infections, and direct operational comparison with simpler targeted approaches before surveillance implementation.

## Data Availability

The datasets generated and analyzed during the current study are available in the NCBI SRA repository under BioProject accession PRJNA1461188.
