# Supplementary material for: Hybridization capture increases on-target nanopore sequencing of plant RNA tobamovirus- derived cDNA libraries
Source: Front Microbiol. 2026 Aug 31;17:1925769. doi: 10.3389/fmicb.2026.1925769 (PMC13572650; doi:10.3389/fmicb.2026.1925769)
Supplement: Supplementary file 1 [file Table_1.docx]

Supplementary Material

# 1 Supplementary Tables

Hybridization capture increases on-target nanopore sequencing of plant RNA tobamovirus-derived cDNA libraries

Andres S. Espindola, Francisco Mosquera-Yuqui, Francisco Ochoa-Corona, and Stephen Marek

Supplementary Table 1. 5′-biotinylated capture probes used for tobamovirus ds-cDNA libraries. Targets are abbreviated as CGMMV (species *Tobamovirus viridimaculae*; cucumber green mottle mosaic virus), PMMoV (species *Tobamovirus capsici*; pepper mild mottle virus) and TMV (species *Tobamovirus tabaci*; tobacco mosaic virus).

| Target | Probe | Sequence (5′–3′) | RefSeq coordinates | GC (%) | Tm (°C) |
| --- | --- | --- | --- | --- | --- |
| CGMMV | CGfmPro1 | GGATTGCTAGGATCTACAACCTCAATGACCCTATTACGCGTATCCGTRGAGCTRAGAAGCGAAACGAAGATRGGCCTCAACACAGGACCGTTGAGGAA | 5973–6070 | 50.0 | 79.4 |
| CGMMV | CGfmPro3 | AATTGGGGYTTYCTCACRCAGATYCCCGACAACGGTAARAYAGCCTTACCYTTYTCTGCCCAGGTGGCAGGTAAAGTCATCGAGGTTC | 2057–2144 | 51.7 | 80.3 |
| PMMoV | PMfmPro1 | TAATTACGTCGTTCGCWAATACACGTCACACRTGTATTWAATTGCGTTCCATCCYAACGACAACCCTTCGATTTAAGTGGAGGGA | 6228–6312 | 42.4 | 78.7 |
| PMMoV | PMfmPro2 | AGTTCTTGCATTGTTGAACTTTTAGCGGACCWGTATACACCATCGCATGCATCTGTTTACGGATAATCGTCTCTGTGGATACCATATGGAACTGCTGCAA | 2101–2200 | 43.0 | 79.6 |
| PMMoV | PMfmPro3 | CATACTTGGAAATGTCYAGCTCTAATATRTCCATAGGWACATYAGAGTCCAGATCTGAGAAAAAYTCTTCGATCTGTGTAGGCGTTTTCCTTG | 4148–4240 | 39.8 | 77.4 |
| TMV | TMfmPro1 | GCGGATGTATATGMACCATATACATTYRACCCGCGCGWTCCAAGACACAACCCTTCGATTTAAGTGGAGGGAAAAACACTATGCGTTATCGTACGCAC | 6283–6364 | 46.4 | 79.2 |
| TMV | TMfmPro2 | ATTCGACACCGCAGCAGATAGTGATGCTACCAGGCTRTCGATAAAGTTTTTCATTTGYTGAACTTTAATCGGACCYGTGTACAC | 2154–2237 | 43.5 | 78.6 |
| TMV | TMfmPro3 | TTATGRACCTCCCATACAGCGTCGTCCAACTGTGTGTAATACGCACAATTGTTCAACGAAMCAGCAACATCACAAAGAGACCTTCTGAACTCCTC | 4725–4819 | 45.3 | 79.3 |

Supplementary Table 2. Anchored reverse primers and template-switching oligonucleotides used for barcoded ds-cDNA library preparation

| Category | Target | Oligo name | Sequence (5′–3′) |
| --- | --- | --- | --- |
| Anchored reverse primer | CGMMV | adap-NBR1-CG | TCTAAGCAGTGGTATCAACGCAGAGTGCGCATATTAGCCTCTGACCAGACTACCG |
| Anchored reverse primer | CGMMV | adap-NBR2-CG | TCTAAGCAGTGGTATCAACGCAGAGTCTGTACCATAGCCTCTGACCAGACTACCG |
| Anchored reverse primer | CGMMV | adap-NBR3-CG | TCTAAGCAGTGGTATCAACGCAGAGTGAACGGTTTAGCCTCTGACCAGACTACCG |
| Anchored reverse primer | CGMMV | adap-NBR4-CG | TCTAAGCAGTGGTATCAACGCAGAGTCGATGTTCTAGCCTCTGACCAGACTACCG |
| Anchored reverse primer | PMMoV | adap-NBR5-PM | TCTAAGCAGTGGTATCAACGCAGAGTAAGCCTGAAAGCGTCTCGGCAGTTGTAG |
| Anchored reverse primer | PMMoV | adap-NBR6-PM | TCTAAGCAGTGGTATCAACGCAGAGTACGAGAACAAGCGTCTCGGCAGTTGTAG |
| Anchored reverse primer | PMMoV | adap-NBR7-PM | TCTAAGCAGTGGTATCAACGCAGAGTTGGAAGCAAAGCGTCTCGGCAGTTGTAG |
| Anchored reverse primer | PMMoV | adap-NBR8-PM | TCTAAGCAGTGGTATCAACGCAGAGTAACAGGTGAAGCGTCTCGGCAGTTGTAG |
| Anchored reverse primer | TMV | adap-NBR9-TM | TCTAAGCAGTGGTATCAACGCAGAGTCGTGTGATGWCCAAACCAAACCAGAAGAGC |
| Anchored reverse primer | TMV | adap-NBR10-TM | TCTAAGCAGTGGTATCAACGCAGAGTTAACGTCGGWCCAAACCAAACCAGAAGAGC |
| Anchored reverse primer | TMV | adap-NBR11-TM | TCTAAGCAGTGGTATCAACGCAGAGTTCGTGCATGWCCAAACCAAACCAGAAGAGC |
| Anchored reverse primer | TMV | adap-NBR12-TM | TCTAAGCAGTGGTATCAACGCAGAGTCAATCAGGGWCCAAACCAAACCAGAAGAGC |
| Template-switching oligo | NS | adap-NBF1-TSO | TCTAAGCAGTGGTATCAACGCAGAGTATATGCGCrGrGrG |
| Template-switching oligo | NS | adap-NBF2-TSO | TCTAAGCAGTGGTATCAACGCAGAGTTGGTACAGrGrGrG |
| Template-switching oligo | NS | adap-NBF3-TSO | TCTAAGCAGTGGTATCAACGCAGAGTAACCGTTCrGrGrG |
| Template-switching oligo | NS | adap-NBF4-TSO | TCTAAGCAGTGGTATCAACGCAGAGTGAACATCGrGrGrG |
| Template-switching oligo | NS | adap-NBF5-TSO | TCTAAGCAGTGGTATCAACGCAGAGTTCAGGCTTrGrGrG |
| Template-switching oligo | NS | adap-NBF6-TSO | TCTAAGCAGTGGTATCAACGCAGAGTGTTCTCGTrGrGrG |
| Template-switching oligo | NS | adap-NBF7-TSO | TCTAAGCAGTGGTATCAACGCAGAGTTGCTTCCArGrGrG |
| Template-switching oligo | NS | adap-NBF8-TSO | TCTAAGCAGTGGTATCAACGCAGAGTCACCTGTTrGrGrG |
| Template-switching oligo | NS | adap-NBF9-TSO | TCTAAGCAGTGGTATCAACGCAGAGTATCACACGrGrGrG |
| Template-switching oligo | NS | adap-NBF10-TSO | TCTAAGCAGTGGTATCAACGCAGAGTCGACGTTArGrGrG |
| Template-switching oligo | NS | adap-NBF11-TSO | TCTAAGCAGTGGTATCAACGCAGAGTATGCACGArGrGrG |
| Template-switching oligo | NS | adap-NBF12-TSO | TCTAAGCAGTGGTATCAACGCAGAGTCCTGATTGrGrGrG |

The 5′ portion of each anchored reverse primer contains a common amplification sequence and an 8-nt barcode followed by the virus-complementary region. Template-switching oligonucleotides contain the same shared 5′ anchor followed by three riboguanosines at the 3′ terminus.

Supplementary Table 3. Oligonucleotides used for RT-qPCR quality control of spiked RNA extracts and duplex qPCR validation of pre- and post-capture ds-cDNA libraries

| Target | Oligo name | Sequence (5′–3′) | Reference |
| --- | --- | --- | --- |
| CGMMV | CGMMVfm7 | GCGTGGTGTTGATCTTACAA | This study |
| CGMMV | CGMMVby77 | CAGAATGCATCCTTGTGTCAA | This study |
| CGMMV | CGMMV-P1 | /5TET/TGTGGTTTC/ZEN/TGGTGTATGGAACGTAC/3IABkFQ/ | This study |
| PMMoV | PMMoVfm10 | GTCCTTTATCTTTGGAGTTTGTCTC | This study |
| PMMoV | PMMoVby10 | CCATTGGAACTGATTCGATGAAC | This study |
| PMMoV | PMMoV-P1 | /5HEX/ACTAGTGTG/ZEN/TCAGAAGGAGGACCC/3IABkFQ/ | This study |
| TMV | TMVfm5 | TACACACAGACAGCTACCAC | This study |
| TMV | TMVby55 | GTAGCAATAAGCGTCTGCTC | This study |
| TMV | TMV-P1 | /56-ROXN/GATCTAGCAAAGCGTCGTCTTTACGA/3IAbRQSp/ | This study |
| NAD5 | nad5-sfm | GATGCTTCTTGGGGCTTCTTGTT | Menzel et al. 2002 |
| NAD5 | nad5-as | CTCCAGTCACCAACATTGGCATAA | Menzel et al. 2002 |
| NAD5 | nad5-P | /56-FAM/AGGATCCGC/ZEN/ATAGCCCTCGATTTATGTG/3IABkFQ/ | Botermans et al. 2013 |
| 18S | 18SF | CGGCTACCACATCCAAGGAA | Aslam et al.  2009 |
| 18S | 18SR | GCTGGAATTACCGCGGCT | Aslam et al.  2009 |
| 18S | 18S-Pfm1 | /56-FAM/CAAATTACCCAATCCTGACACGGGGAGG/3IABkFQ/ | This study |

NAD5 was retained as the initial plant control assay for extract-level QC. After screening four candidate host assays (ACTIN, GAPDH, EF1α, and 18S), 18S was selected for the final duplex qPCR validation because it was the only host assay that consistently amplified in retained post-capture ds-cDNA libraries together with the virus-specific assays.

Supplementary Table 4. Average library sizes estimated from fragment analysis

| Target | Replicate | Pre-capture size (bp) | Post-capture size (bp) |
| --- | --- | --- | --- |
| CGMMV | 1 | 1200 | 1400 |
| CGMMV | 2 | 1400 | 2100 |
| CGMMV | 3 | 1400 | 1750 |
| CGMMV | 4 | 2300 | 2800 |
| PMMoV | 1 | 1500 | 1500 |
| PMMoV | 2 | 1500 | 1500 |
| PMMoV | 3 | 1100 | 1200 |
| PMMoV | 4 | 1200 | 1200 |
| TMV | 1 | 1400 | 1400 |
| TMV | 2 | 1800 | 2000 |
| TMV | 3 | 1800 | 2500 |
| TMV | 4 | 1500 | 2200 |

Supplementary Table 4 lists the 12 pre-/post-capture library pairs retained for the final sequencing comparison (four per virus-host combination). Additional libraries generated during assay development were not carried forward when outlier fragment profiles or poor post-capture recovery prevented matched analysis.

Supplementary Table 5. Standard-curve performance for duplex qPCR assays used to validate pre- and post-capture ds-cDNA libraries

| Virus assay | Target quantified | Channel | Paired libraries (n) | Slope | Efficiency (%) | R² |
| --- | --- | --- | --- | --- | --- | --- |
| CGMMV | CGMMV | A.Yellow | 5 | -3.437 | 95.4 | 0.9996 |
| CGMMV | 18S | A.Green | 5 | -3.454 | 94.8 | 0.9997 |
| PMMoV | PMMoV | A.Yellow | 6 | -3.439 | 95.3 | 0.9998 |
| PMMoV | 18S | A.Green | 6 | -3.450 | 94.9 | 0.9995 |
| TMV | TMV | A.Orange | 4 | -3.573 | 90.5 | 0.9989 |
| TMV | 18S | A.Green | 4 | -3.592 | 89.8 | 0.9976 |

Standard curves were estimated by linear regression of Ct against log10-transformed gBlock concentration over a six-point 10-fold dilution series from 0.05 to 5 × 10−7 ng/µL.

Supplementary Table 6. Ct values and normalized virus-to-18S changes in paired pre- and post-capture ds-cDNA libraries

| Virus assay | Pair | Pre-capture library | Post-capture library | 18S Ct (pre / post) | Virus Ct (pre / post) | Virus/18S ratio (pre / post) | Post/pre fold change |
| --- | --- | --- | --- | --- | --- | --- | --- |
| CGMMV | 1 | CCG1pre | HCG1pos | 17.68 / 15.54 | 18.82 / 11.55 | 0.4 / 12.5 | 31.25 |
| CGMMV | 2 | CCG2pre | HCG2pos | 18.49 / 16.30 | 18.72 / 11.35 | 0.733 / 23.6 | 32.20 |
| CGMMV | 3 | CCG3pre | HCG3pos | 18.75 / 14.24 | 20.63 / 12.34 | 0.241 / 3.08 | 12.79 |
| CGMMV | 5 | CCG5pre | HCG5pos | 18.79 / 16.41 | 20.00 / 13.30 | 0.379 / 6.86 | 18.13 |
| CGMMV | 6 | CCG6pre | HCG6pos | 19.69 / 17.23 | 18.48 / 12.26 | 1.91 / 23.8 | 12.49 |
| PMMoV | 7 | PPM7pre | HPM7pos | 19.50 / 19.34 | 16.09 / 15.60 | 7.73 / 9.68 | 1.25 |
| PMMoV | 8 | PPM8pre | HPM8pos | 21.37 / 20.59 | 14.07 / 10.62 | 105 / 622 | 5.93 |
| PMMoV | 9 | PPM9pre | HPM9pos | 17.36 / 14.37 | 16.36 / 15.10 | 1.55 / 0.49 | 0.32 |
| PMMoV | 10 | PPM10pre | HPM10pos | 20.69 / 18.95 | 15.31 / 12.08 | 29 / 78.4 | 2.70 |
| PMMoV | 11 | PPM11pre | HPM11pos | 19.31 / 17.54 | 14.99 / 12.43 | 14.3 / 24.3 | 1.70 |
| PMMoV | 12 | PPM12pos | HPM12pos | 21.80 / 21.20 | 14.96 / 10.01 | 77.1 / 1.41e+03 | 18.32 |
| TMV | 13 | TTM13pre | HTM13pos | 19.21 / 15.83 | 26.89 / 24.39 | 0.0342 / 0.0196 | 0.57 |
| TMV | 15 | TTM15pre | HTM15pos | 18.07 / 16.20 | 26.16 / 26.00 | 0.0264 / 0.00882 | 0.33 |
| TMV | 16 | TTM16pre | HTM16pos | 20.10 / 18.97 | 27.81 / 27.45 | 0.0335 / 0.0205 | 0.61 |
| TMV | 17 | TTM17pre | HTM17pos | 20.66 / 17.68 | 29.70 / 27.47 | 0.0142 / 0.00884 | 0.62 |

Virus/18S ratios were calculated from the corresponding channel-specific standard curves. In the PMMoV Rotor-Gene export, the library labeled PPM12pos corresponds to the confirmed pre-capture mate of HPM12pos.
